# Perspective on the current state of the LRRK2 field

**DOI:** 10.1038/s41531-023-00544-7

**Published:** 2023-07-01

**Authors:** Jean-Marc Taymans, Matt Fell, Tim Greenamyre, Warren D. Hirst, Adamantios Mamais, Shalini Padmanabhan, Inga Peter, Hardy Rideout, Avner Thaler

**Affiliations:** 1 Univ. Lille, Inserm, CHU Lille, UMR-S 1172—LilNCog—Lille Neuroscience & Cognition, F-59000 Lille, France; 2 Merck & Co., Inc., 33 Avenue Louis Pasteur, Boston, MA 02115 USA; 3 Pittsburgh Institute for Neurodegenerative Diseases, University of Pittsburgh, 3501 Fifth Avenue, Suite 7039, Pittsburgh, PA 15260 USA; 4 Neurodegenerative Diseases Research Unit, Biogen, 115 Broadway, Cambridge, MA 02142 USA; 5 Center for Translational Research in Neurodegenerative Disease, Department of Neurology, University of Florida, Gainesville, FL USA; 6 The Michael J. Fox Foundation for Parkinson’s Research, Grand Central Station, P.O. Box 4777, New York, NY 10120 USA; 7 Department of Genetics and Genomic Sciences, Icahn School of Medicine at Mount Sinai, 1425 Madison Ave, New York, NY 10029 USA; 8 Centre for Clinical, Experimental Surgery, and Translational Research, Biomedical Research Foundation of the Academy of Athens, Athens, Greece; 9 Movement Disorders Unit and Laboratory of Early Markers of Neurodegeneration, Neurological Institute, Tel-Aviv Medical Center, Faculty of medicine, Tel-Aviv University, Tel-Aviv, Israel

**Keywords:** Parkinson's disease, Parkinson's disease

## Abstract

Almost 2 decades after linking *LRRK2* to Parkinson’s disease, a vibrant research field has developed around the study of this gene and its protein product. Recent studies have begun to elucidate molecular structures of LRRK2 and its complexes, and our understanding of LRRK2 has continued to grow, affirming decisions made years ago to therapeutically target this enzyme for PD. Markers of LRRK2 activity, with potential to monitor disease progression or treatment efficacy, are also under development. Interestingly, there is a growing understanding of the role of LRRK2 outside of the central nervous system in peripheral tissues such as gut and immune cells that may also contribute to LRRK2 mediated pathology. In this perspective, our goal is to take stock of LRRK2 research by discussing the current state of knowledge and critical open questions in the field.

## Introduction

**Fig. 1:**
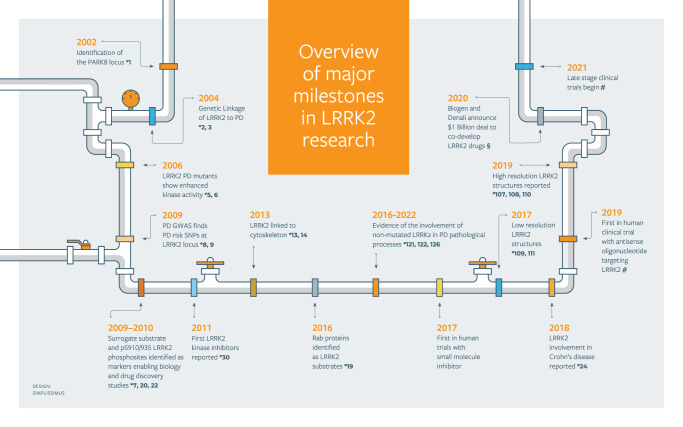
Timeline of key milestones in the advancement of the field of LRRK2 research. Numbers next to the stars (*) denote the citation references pertaining to the milestone. #See text above for the ClinicalTrials.gov identifiers (NCT numbers) for the clinical milestones. §https://investors.biogen.com/news-releases/news-release-details/biogen-and-denali-collaborate-lrrk2-program-parkinsons-disease.

The *leucine-rich repeat kinase 2* (*LRRK2*) gene was identified in 2004 as the gene responsible for the PARK8 locus that had itself previously been linked to Parkinson’s disease (PD) in 2002^1–3^ (see timeline of key milestones in the LRRK2 field in Fig. 1). Almost 20 years later, a PubMed search using LRRK2 as search term results in >3000 publications and a thriving research field has developed around the study of LRRK2. One of the reasons for this is that from the moment of its discovery LRRK2 showed that it was potentially a major determinant in the pathophysiology of PD. The study of neurodegenerative diseases of aging are a priority area for research worldwide, as the number of individuals with these diseases is dramatically increasing due to the aging population, and LRRK2 has received significant attention. What research on LRRK2 has revealed thus far confirms that this attention is well deserved and may ultimately lead to improvements in the diagnosis of PD and novel therapies for the patients.

**Fig. 2:**
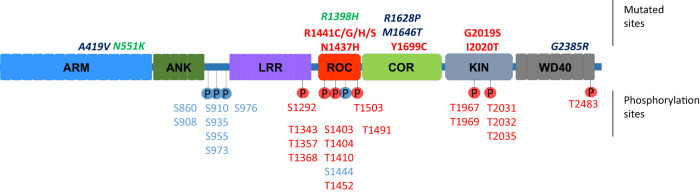
Schematic of the LRRK2 domain structure. Above the schematic, key amino acid substitutions are indicated that alter risk for PD, including mutations that increase risk for PD such as pathogenic mutations (red) and risk factor mutations (blue) as well as mutations that confer reduced risk for PD (green). Phosphorylated residues are given below the schematic, with heterologous phosphosites give in blue and autophosphorylation sites in red. Figure adapted from ref. ^22^.

The *LRRK2* gene is located on chromosome 12, contains 51 exons spanning 144 kb and encodes for a large 286 kDa multidomain protein of the same name. As its name suggests, LRRK2 contains a leucine-rich repeat (LRR) domain and a kinase domain. LRRK2 was identified as a member of the Ras of complex proteins (ROCO) family^4^ and thus it also contains a Ras-like GTPase domain (ROC) followed by a C-terminal of ROC (COR) domain. In addition, sequence homology revealed the presence of additional domains, including an armadillo repeat domain (ARM) and ankyrin repeat domain (ANK) in the N-terminus and a WD40-repeat at the C-terminus (Fig. 2). In the first years after its discovery, work on *LRRK2* genetics revealed several missense mutations that segregated with disease, many located in LRRK2’s catalytic core, constituted of the ROC, COR and Kinase domains. Mutations had an autosomal dominant mode of transmission and when biochemical analysis showed that many mutations led to an increase in LRRK2 autophosphorylation activity^5,6^ as well as an increase in phosphorylation of surrogate substrates^7^, the notion of a gain of toxic kinase function was born to explain LRRK2’s pathophysiological mechanism(s) in PD. Although LRRK2 mutations were found to be relatively common compared to mutations in other PD genes, these are still only affecting a small proportion of all PD patients suggesting *LRRK2* may only be a relevant gene for a subset of patients^8^. However, in 2009 and following, PD genome-wide association studies (GWAS) identified common genetic variance at and near the *LRRK2* locus as a risk factor for PD^9,10^, pointing to the possibility that LRRK2 dysfunction may be involved in PD pathomechanisms in patients who do not harbour a missense mutation. These findings of genetic evidence that LRRK2 is involved in PD and that the kinase hyperactivation may be a primary culprit were strong drivers of research programmes in academia and industry to understand LRRK2 dysfunction and develop inhibitors of LRRK2 kinase activity.

Studies on LRRK2 have extensively explored the toxic gain of function hypothesis for LRRK2 suggesting that expression of a disease mutant form of LRRK2 would lead, in cellular or in vivo models, to PD phenotypes, notably cell death, the presence of intracellular inclusions or loss of synaptic connections. However, research has shown that LRRK2 mutants are relatively well tolerated, in cells and in animals, and they do not induce the severe disease phenotypes that were predicted (reviewed in^11,12^). What has emerged through these efforts are specific cellular functions that are modulated by LRRK2 activity. For instance, several reports have described the involvement of LRRK2 in the cytoskeleton, including interactions with microtubules or actin^13,14^, as well as the modulation of cell morphology^15^. Another series of reports points to a role of LRRK2 in the endolysosomal system^16–18^. One key finding in this regard is that a subset of Rab proteins are physiological substrates of LRRK2^19^, coinciding with several observations showing the involvement of LRRK2 in intracellular trafficking or endolysosomal functions, such as its recruitment to lysosomes or Golgi apparatus or its regulation of receptor trafficking^20^. Another major feature of LRRK2 is that it is multiphosphorylated (Fig. 2), including heterologous phosphorylation sites and autophosphorylation sites that are observed to be inversely regulated in disease (reduction for the heterologous sites clustering around S910/S935^21^ and increase for the autophosphorylation sites^5,6^) while both are reduced after pharmacological treatments with type I kinase inhibitors^22,23^. Another significant development over the past decade is the increased understanding that LRRK2 is important in several peripheral functions such as the immune system or the gut, and that these functions may also contribute to the PD pathomechanisms. Examples of this are the observations that LRRK2 expression and phosphorylation is upregulated in B-cells and microglia upon immune challenge and the identification of disease linked mutations for LRRK2 in Crohn’s disease^24^, a type of immune-mediated inflammatory bowel disease, as well as observations that pathological α-synuclein recruits LRRK2 expressing pro-inflammatory monocytes from the periphery to the brain^25^. Several recent reviews discuss in more detail the roles of LRRK2 in the periphery^26–29^.

In terms of LRRK2 as a therapeutic target, several inhibitors of the LRRK2 kinase have been developed in the past decade or more, including initial non-brain penetrant compounds LRRK2-IN1^30^ and GSK2578215A^31^, followed by several brain penetrant compounds GNE7915, GNE-0877^32^, PF-475^33^, PF-360^34^, MLi-2^35^, DNL201^36^, and antisense oligonucleotides^37^, the best of which demonstrate potency in the low nanomolar range and very good selectivity. These tools have allowed the advancement of LRRK2 kinase inhibitors through preclinical testing, with thee small molecule inhibitors (DNL201, DNL151, ClinicalTrials.gov Identifiers NCT04557800, NCT05119790, NCT04056689, NCT05005338, NCT05418673, NCT05348785, NCT05229562, NCT04551534, NCT03710707, and NEU-723 ClinicalTrials.gov Identifier NCT05633745, note BIIB122 is an alternate name for DNL151) and one anti-sense oligonucleotide (BIIB094, ClinicalTrials.gov Identifier NCT03976349) in clinical trials as potential treatments for PD.

The research around LRRK2 also led to initiatives to facilitate discussion and scientific exchanges, such as the creation, in 2008, of a specific international LRRK2 consortium hosted by the Michael J. Fox Foundation and the organisation of an International Scientific Conference on LRRK2 in 2012 by the Biochemical Society. Both of these exchange initiatives are still active, in the form of a PD Research Exchange forum (including LRRK2 as well as several other areas of PD research) and a Biennial International LRRK2 conference, the fourth and latest edition of which was held in June-July of 2022. In this paper, our goal is to take stock of the current state of advances in the field of LRRK2 research by discussing current key questions in the field and what perspectives these point to for the future.

### What is the normal vs pathological function of LRRK2?

Since the identification and implication of LRRK2 in PD, much attention has been paid to how LRRK2 pathogenic mutations alter specific functions of this enzyme relative to the wild type LRRK2, while not yet having a detailed picture of the normal function of LRRK2. Hence, a further understanding of the phenotypes associated to the loss of LRRK2 for instance by the study of LRRK2 KO animals under basal conditions, has proven insightful. Indeed, as described above, contours are emerging of the normal function of LRRK2, however several questions remain. For instance, is LRRK2 an agent of the cytoskeleton, or an agent of the endolysosomal system? Primary neurons isolated from LRRK2 KO mice are reported to develop longer and more complex neurites^14^, suggesting a role for LRRK2 in the cytoskeleton and cell morphology. By contrast, several findings point to a role of LRRK2 in membrane trafficking or subcellular organization, such as the ability of LRRK2 to phosphorylate several members of the family of Rab-GTPases^19^, the ability of LRRK2 to be recruited to specific subcellular compartments such as Golgi network (via Rab29 overexpression)^38^ or lysosomes (under influence of lysosomotropic agents)^39^. The link between LRRK2 and Rabs are potentially also relevant to a role for LRRK2 in centrosome cohesion^40^ and ciliogenesis^41,42^. Besides involvement in the cytoskeleton and in membrane trafficking, additional cellular functions are reported to be affected by LRRK2, including iron homoeostasis^43^ and mitochondrial function^44^.

Aside from normal function, work with LRRK2 deficient models would also be helpful in discerning whether native unmutated LRRK2 may contribute to disease. Mice and rat LRRK2 knockouts (KO) are viable and do not display strong changes in outward signs of health^45–47^. Although initial work did not demonstrate changes in toxicity induced in transgenic α-synuclein overexpression or MPTP in LRRK2 KO animals^45,48^, several other studies report results consistent with the conclusion that LRRK2 is a modulator of toxicity. Some relevant examples are reports that toxicity induced by nigral α-synuclein overexpression, LPS, HIV-1 Tat or *Mycobacterium tuberculosis* in rodent models is abrogated in LRRK2 KO animals^49–51^. If LRRK2 is a toxicity modifier, is this the primary toxic mechanism for LRRK2 or might LRRK2 also act as a toxic agent in its own right?

The study of LRRK2 KO animals may also help in answering additional key questions, such as is the toxic function of LRRK2 in the brain or in the periphery? Observations of LRRK2 KO animals show that peripheral tissues such as kidneys or lungs display a microvacuolation phenotype that is not observed in the brain^47^. Another phenomenon observed in the periphery and not in the CNS is that LRRK2 KO mice and rats show an age dependent darkening of the kidneys^46^, a phenomenon which is reported to be linked to haemoglobin and lipofuscin accumulation in renal tubules^52^ although little is known on how this may be involved in pathological processes. LRRK2 is also expressed in the gastrointestinal (GI) tract^53^ and LRRK2 KO mice show reduced symptoms in an experimental colitis model^54^, suggesting that LRRK2 affects GI pathologies. Further, taking these data together with the findings that patients with Crohn’s disease (inflammatory bowel disease, IBD) have a higher risk of developing PD (meta-analysis^55^) may also point to a role for LRRK2 in the GI in the development of PD. Other peripheral tissues/systems are also impacted in LRRK2 KO animals as observed by vacuolation of type-2 pneumocytes in the lungs^34,56^ or altered function of immune cells^57,58^. Therefore, the question arises: should we be looking more at immune function and GI function of LRRK2 rather than brain function?

Besides the fundamental scientific goal of fully understanding LRRK2, improving our understanding of the normal function of LRRK2 may teach us how this is altered in PD. This, in turn, would point to possibilities to identify and stratify patients, among others by the identification of LRRK2 based biomarkers of disease in urine, blood, CSF or other patient based samples^59^. Developing the potential of the LRRK2 pathway for use as biomarkers (examples given below in the section on therapeutics) would be a welcome addition to the panel of potential measures included in clinical testing, given the concern that trials may fail because we do not have the right measures of LRRK2 disease-involvement in an individual or of target engagement in those receiving targeted therapy.

### What about LRRK2 mutations and penetrance?

For many diseases with genetic causes, the simple presence of a disease linked genetic mutation does not coincide 100% with the presence of the disease. This is also the case for LRRK2 (an overview of LRRK2 mutations in given in Table 1). For instance, many individuals carrying the LRRK2 G2019S mutation will never develop PD, even at very old ages^60,61^. The estimates of the lifetime penetrance of LRRK2 G2019S is wide, from 17 to 80%^62–64^, for instance 25% at the age of 80 among Ashkenazi Jews^62^ and around 40% among non-Ashkenazi Jews^65^. Other mutations, such as G2385R, may have even lower penetrance, for example: 10% at 80 years of age^63,66^, yet early estimates of R1441G penetrance were higher, reaching 80% at 80 years of age^67^ with a potentially more homogenous phenotype^68^, which could be important for clinical trials. These observations beg the question: why do some individuals carrying LRRK2 mutations develop PD and others do not? Which additional factors contribute to the penetrance of LRRK2 mutations and are they genetic or are they environmental? There may in fact be evidence for both. For instance, caffeine consumption is observed to have a greater protective effect in LRRK2 G2019S carriers compared to non-mutation carriers^69^. Also, genetic epistasis has been observed between risk SNPs at the *PARK16* (with RAB29 as nominated gene responsible for this locus) and *LRRK2* loci^70^, and LRRK2 G2019S penetrance is influenced by a polygenic risk score^71^.

**Table 1:** Overview of LRRK2 amino-acid substitutions that alter disease risk.

Mutation	Domain where mutation is located	Nature of the mutation	Reported prevalence in specific populations
A419V	ARM	Increased risk for PD	East Asian
N551K	ARM	Reduced risk for PD	East Asian, Ashkenazi Jewish, non-Jewish European
R1398H	ROC	Reduced risk for PD	European, East Asian, Ashkenazi Jewish, non-Jewish European
N1437H	ROC	Increased risk for PD	European
R1441C	ROC	Increased risk for PD	European
R1441G	ROC	Increased risk for PD	European Basque
R1441H	ROC	Increased risk for PD	European
R1628P	COR	Increased risk for PD	East Asian
Y1699C	COR	Increased risk for PD	European
G2019S	Kinase	Increased risk for PD	European/West Asian, Mixed populations
I2020T	Kinase	Increased risk for PD	East Asian
N2081D	Kinase	Increased risk for CD and potentially PD	Ashkenazi Jewish, non-Jewish European
G2385R	WD40	Increased risk for PD	East Asian
M2397T	WD40	Increased Risk for CD and Leprosy	East Asian, Mixed populations

*PD* Parkinson’s disease, *CD* Crohn’s disease (data in the table from references ^24,72,73^).

While there are several LRRK2 pathogenic mutations associated with PD, G2019S LRRK2 has been extensively studied given the prevalence of this mutation over other disease-causing mutations^64^. The clinical phenotype of G2019S LRRK2-PD can be described as a typical PD syndrome with both motor and non-motor symptoms and a clear response to levodopa. However, several unique characteristics have emerged: a higher percent of gait manifestations and lower extremity onset^72,73^ and slower motor progression^74^. Better cognitive performance^75^ and slower cognitive decline compared to sporadic cases^74^ have been described as well. In addition, less non-motor involvement including lower rates of hyposmia and REM sleep behaviour disorder^75–77^ have been reported. While some structural imaging studies managed to detect increased cerebral volume among LRRK2-PD compared with iPD^78^, others did not and reported decreased cortical thickness between LRRK2-PD and healthy controls, with no difference between LRRK2-PD and iPD^79^. Dopamine transporter SPECT studies indicate higher striatal binding ratios (SBR) in the contralateral caudate and putamen among LRRK2-PD compared with iPD^80^. Interestingly, while the age of onset of PD in LRRK2 G2019S carriers is comparable to sporadic PD, the disease progression of LRRK2 G2019S PD patients is slower than sporadic PD^74,81,82^. Thus, it seems that LRRK2-PD has a more favourable clinical phenotype^83^ and perhaps even longer survival than iPD patients^84,85^. While these group differences remain to be defined unambiguously, it should be emphasized that these clinical phenotypes cannot distinguish, on an individual basis, G2019S from sporadic cases. Interestingly, mutations in the GBA gene are the strongest risk factors for PD^86^, and like the G2019S mutation in the LRRK2 gene, are common among Ashkenazi Jews. Harbouring a mutation in the GBA gene has been associated with a more severe and rapidly deteriorating phenotype of PD^87,88^. Yet, dual mutation carriers (GBA-LRRK2-PD) have been found to have a milder disease phenotype compared to GBA-PD^89–91^, raising the possibility of a protective mechanism for LRRK2 on the GBA disease phenotype.

These observations raise a controversial point: if LRRK2-PD subjects do indeed have a milder phenotype than iPD, then perhaps treating LRRK2 might worsen instead of improving the disease. It remains to be demonstrated whether the LRRK2 mutation status of a patient changes the potential benefit of these patients for specific PD therapies and it will certainly be necessary to factor in this aspect when planning clinical studies and analysing clinical study data.

### LRRK2 pathology: distinct from idiopathic PD?

In pathological studies performed to date in LRRK2-PD, variable changes have been described including Lewy bodies, tau positive inclusions, TDP-43 aggregates, ubiquitin inclusions and pure nigral cell loss with no inclusions^92,93^. Recent advances in aggregation assays have enabled the assessment of α-synuclein from cerebrospinal fluid (CSF) and other tissues from patients with PD. LRRK2-PD carriers have been found to have lower rates of α-synuclein seed amplification on these assays as compared to both idiopathic PD and GBA-PD^94,95^. These differences in pathological findings, together with the clinical and imaging studies, could explain the existence of different subgroups within LRRK2-PD^93^. In fact, a recent study assessing olfactory functions in LRRK2-PD identified a subgroup of patients with earlier age of onset and faster olfactory loss^76^. Multi-omics profiling of human biofluids from LRRK2 mutation carriers are beginning to identify additional pathways that are altered in LRRK2-PD and LRRK2 non-manifesting carriers when compared to iPD and healthy controls^96,97^. These data suggest that multiple mechanisms may exist in LRRK2 carriers that lead to PD pathogenesis and progression. Translating our understanding of these mechanisms into better biomarkers to diagnose at-risk individuals, monitor progression and enrich patients for clinical trials will be critical- especially given the heterogeneity in clinical, pathological and biological endpoints in LRRK2 cohorts. Future studies should be aimed at correlating biological endpoints with clinical, imaging and pathological measures of LRRK2-PD to better understand the biological basis of the subgroups within LRRK2. Such analyses will be critical for patient selection into clinical trials as different LRRK2 mutation carriers may benefit from different treatment strategies^98^.

It is noteworthy that with distinct LRRK2 mutations, I2020T^99^ and G2019S^93,100,101^, the reported pathology differs from idiopathic PD. In the I2020T mutation carriers there was dopaminergic neuron loss in the substantia nigra in 6 of the 8 cases, with no Lewy bodies, nor other marked pathology e.g. Tau^99^. The first case study with G2019S^100^ showed no Lewy bodies but did show tau-immunoreactive neurofibrillary tangles and an argyrophilic grain disease–like pathology. More recently Kalia et al. showed that 30% of G2019S mutations carriers and 70% of other mutations carriers (including I2020T, R1441C, R1441G and Y1699C) did not have Lewy bodies, but did have dopaminergic neuron degeneration^93^. Kalia et al. did not report on Tau pathology, but this has been addressed by Henderson et al.^101^. These investigators found that α-synuclein pathology was present in 63.6% of LRRK2 mutation carriers, but tau pathology was found in 100% of carriers and is abundant in 91% of carriers. They used an antibody which selectively binds Alzheimer’s disease (AD)-type tau and used quantitative analysis of this tau pathology to demonstrate that AD tau is the prominent type of tau present in LRRK2 mutation carriers. They also make the important point that AD tau staging in LRRK2 PD follows a similar distribution to iPD and iPD with dementia and is accompanied by abundant concurrent Aβ pathology in most cases. They suggest that tau is not an independent disease factor in LRRK2 PD, but is associated with the degree of α-synuclein pathology and progression to dementia^101^. Moreover, there is recent data that goes one step further, suggesting that the initiation of dopaminergic neurodegeneration occurs independently of α-synuclein aggregation and is likely tau mediated^102^. The lower incidence of α-synuclein pathology has recently been addressed in ante-mortem CSF samples using α-synuclein seeding amplification assays^94,103^, where positive signal was observed in only 40–78% of LRRK2 mutation carriers. However, this may be dependent on the techniques used and the forms of α-synuclein measured (Sekiya et al. 2022, Ann Neurol, 92: S1-S243, M177. 10.1002/ana.26484). These studies all point to subgroups of LRRK2 PD patients based on whether they harbour α-synuclein or Lewy body deposits.

Overall, the prevalence of Tau pathology and the reduced incidence of pronounced α-synuclein pathology in certain LRRK2 mutation carriers does raise important considerations with respect to inclusion criteria in clinical trials, endpoints (biomarkers) measured and ultimately the therapeutic strategies. It could be beneficial to stratify the mutation carriers based on these pathologies with respect to future clinical trials, particularly if additional data were to emerge that provides clearer links between disease progression and one or more co-pathologies. The availability of Tau imaging agents^102^ and the potential utility of blood-based assays to measure specific Tau species^104^, could enable this, indeed there are already efforts to image Tau in G2019S subjects (ClinicalTrials.gov Identifier: NCT04557865).

The ongoing clinical trials will hopefully be able to perform *post-hoc* analysis of their data to increase our understanding of this complex interplay between LRRK2 mutations and pathophysiology. Future trials, using combination therapies e.g. reducing LRRK2 and Tau, may be required to alter disease progression, employing a precision medicine strategy enabled by biomarkers.

### LRRK2 as a therapeutic target

LRRK2 is a therapeutic target for Parkinson’s disease (PD). Indeed, findings of LRRK2 hyperactivation in disease mutants and that deleterious effects of LRRK2 mutants in preclinical models can be blocked by inhibiting LRRK2 expression or kinase activity provided an initial validation of LRRK2 as a therapeutic target^105,106^. Efforts to develop LRRK2 targeting agents have met a certain degree of success as potent and selective agents targeting LRRK2 have been developed, both small molecule inhibitors of LRRK2 kinase activity and antisense oligonucleotides to reduce LRRK2 levels, and these targeting approaches are currently under evaluation in early phases of clinical trials. While this is very encouraging in the path to PD modifying therapies, it should be noted that the end goal of validating LRRK2 as a PD therapeutic target has yet to be reached. For validation of a therapeutic target, it is necessary to demonstrate significant and reproducible disease modification in human patients following pharmacological targeting, i.e. after successful phase III clinical trials. One of the potential pitfalls in this process is that specific targeting agents are selected to be evaluated, and it may be that these display shortcomings or secondary effects that can lead to inconclusive clinical results. It is therefore important to test more than 1 agent and/or more than one targeting modality for a given target.

The study of LRRK2 biology has revealed that LRRK2 itself can be targeted in more ways than one. Besides LRRK2 kinase activity, LRRK2 function is also regulated by its GTPase domain and its quaternary structure, both of which are potentially targetable. We have also advanced in our understanding of key cellular partners of LRRK2 that participate in generating the ‘output’ of the LRRK2 pathway that is deregulated in disease. In as far as these partners are crucial to the functioning of the LRRK2 pathway, they may constitute valuable therapeutic targets for PD in their own right.

Recent advances in the elucidation of the 3D domain organisation and atomic structure of LRRK2^107–111^ are a very big boost to the field on several levels. For instance, we can now integrate rational design steps in the therapeutic development process. This can be at the level of molecules targeting catalytic pockets of LRRK2 enzymatic functions, as well as to understand molecules targeting at allosteric sites or even at sites that would mediate the quaternary structure or protein-protein interactions with LRRK2 partners in the event of the design of protein-protein interaction modulators. The potential of pursuing additional 3D structural information for LRRK2 is big. Indeed, structures that have been identified thus far are reflecting LRRK2 in specific states depending on the experimental conditions. By studying additional conditions such as pH, binding partners or mutations, additional structures are likely to emerge, that will shed light on the structural dynamics of LRRK2. It should also not be excluded that specific conformations of LRRK2 may be identified that are reflective of healthy or pathological states^112^. In this case, this phenomenon may be useful as a screening tool for compounds promoting a healthy conformation, but also can open a path for developing conformation specific probes for use in biomarker testing. Conformation specific antibodies have already been developed to identify different α-synuclein species, suggesting a similar approach could be feasible for LRRK2^113^.

Concerning the targeting strategies themselves, LRRK2 kinase inhibition has taken a very significant lead ahead of other targeting options for LRRK2. Undoubtedly, this was influenced by significant existing expertise in drug discovery organisations on developing kinase inhibitors. Those inhibitors that have been most extensively tested are type I inhibitors, i.e. inhibitors that bind to the active conformation of the kinase. Interestingly, recent data suggests that this class of inhibitor is responsible for inducing the recruitment of LRRK2 to microtubules^107^. These same inhibitors may display a potential unwanted side effect as they are reported at high doses to generate a vacuolation phenotype in kidneys and type 2 pneumocytes upon chronic treatment in rodents and non-human primates^34,56^. Understanding the link between type I kinase inhibitors of LRRK2 and these undesired phenotypes would be most useful to pre-empt potential failure of this class of compounds. Alternative solutions could be to develop type II kinase inhibitors for LRRK2, i.e. inhibitors that target and stabilize an inactive form of LRRK2 or a knock down approach using anti-sense oligonucleotides (ASO) to LRRK2 that are in clinical development (ClinicalTrials.gov Identifier: NCT03976349) with intrathecal administration. The ASOs are poorly taken up by type 2 pneumocytes and may provide differentiation from the small molecules if the lung phenotype proves to be an issue. Further work will be needed to determine whether targeting the LRRK2 kinase or expression is indeed sufficient and/or required to stop disease without strong side effects.

By analogy with the questions on LRRK2 function in the CNS compared to peripheral tissues, it is yet to be determined whether targeting LRRK2 in the periphery would be beneficial or detrimental. While the vacuolation observed in the kidneys or lungs in certain conditions after LRRK2 kinase inhibition is not considered as a protective marker, it can be hypothesized that targeting LRRK2 in the GI tract or immune system may be protective, for instance by positively affecting intestinal inflammation^114^ or by blocking transmission of toxic α-synuclein species from the GI tract to the CNS through the vagus nerve. In addition, further work is warranted to verify whether therapeutic targeting should be focused specifically for the central nervous system or whether targeting of LRRK2 in periphery should be preferred, or whether it is best to target both central and peripheral LRRK2.

### Treatment of iPD with LRRK2 targeting agents?

Aside from PD-causative mutations, PD risk has also been identified in genomic variants at the LRRK2 locus, raising the possibility that LRRK2 may be involved in the pathogenesis of iPD independent of coding mutations. Given that pathogenic mutations of LRRK2 are associated with aberrantly enhanced kinase activity and, consequently, that kinase inhibition may be useful therapeutically, it is worth investigating the LRRK2 kinase activation state in iPD. In this context, preclinical rodent models showed that genetic ablation or pharmacological kinase inhibition of endogenous wild-type LRRK2 protects against α-synuclein-induced neurodegeneration^49^. Implicit in such a conclusion is the assumption that wild-type LRRK2 kinase must impact nigrostriatal dopamine neurons under these experimental conditions. Similarly, in an ‘environmental’ model of PD in wild-type rats, it was demonstrated that exposure to rotenone (which increased LRRK2 kinase activity as assessed by pThr73-Rab10 levels) could reproduce endolysosomal deficits seen in human post-mortem brains—and treatment with a LRRK2 kinase inhibitor could prevent them and thereby avert α-synuclein pathology and associated neurodegeneration^115^.

Of crucial relevance to starting LRRK2 targeting therapies in general and for iPD in particular, is the availability of biomarker tests to probe the activity of the LRRK2 pathway in clinical samples. Several assays exist for this, primarily measuring auto- and substrate phosphorylation to assess LRRK2 kinase activity or measuring LRRK2 phosphorylation at heterologous sites known to impact LRRK2 subcellular distributions and complex formation (reviewed in Rideout et al.^59^). For instance, by measuring levels of the autophosphorylation site, pSer1292-LRRK2, and the LRRK2 substrate, pThr73-Rab10, it was demonstrated that LRRK2 kinase activity is elevated in nigrostriatal dopamine neurons in post-mortem brain tissue from individuals with iPD^116^. Moreover, a recent study developed a cytometry based assessment of peripheral blood mononuclear cells (PBMCs) for LRRK2, Rab and GBA, and observed elevated levels of LRRK2 and phosphorylation of Rab10 in PBMC samples from subjects with iPD^117^. In urinary extracellular vesicles (EVs), several changes have also been observed in patients. Increased pSer1292-LRRK2 in urinary EVs has been observed in specific iPD cohorts^118,119^, although additional studies did not replicate this^120^, suggesting that this increase may be linked to a subset of patients. In addition, reduced phosphorylation of the heterologous phosphosites S910-LRRK2 and S935-LRRK2 and increased Rab8 and EV abundance is observed in urinary EVs in iPD^120^. There is still a gap in the data with respect to CSF measurements of (elevated) pRabs in disease (idiopathic and mutation carriers), which may be, at least in part, due to technical limitations in the measurements of the phosphorylated Rab proteins or in the processing of this sample type. Thus, there is abundant experimental and human data indicating that LRRK2 kinase activity is abnormally increased in iPD and models thereof—and the experimental studies strongly suggest that blocking LRRK2 kinase may be neuroprotective. It should also be noted that a PD risk SNP at the *LRRK2* locus has been associated with dysregulated microglial function^121^.

In sum, these results implicate wild-type LRRK2 in iPD and, for the most part, indicate that LRRK2 kinase activity may be a viable therapeutic target. However, unanswered questions remain. For example, since pre-clinical LRRK2 therapeutics have generally only been tested before or coincident with the toxic insults that form the basis of parkinsonian models, it remains to be determined whether such strategies will also be effective if started once the degenerative process is already well underway, such as at the time of clinical diagnosis of PD.

Another outstanding question in the field is what level of LRRK2 kinase inhibition is required to drive efficacy in the clinic, especially as it pertains to treating patients with iPD. In LRRK2 mutation carriers, the elevation in kinase activity is ~2–4 fold higher than non-mutation carriers, thus is has been hypothesised that inhibiting LRRK2 kinase activity by ~50% would effectively normalise the overactive kinase activity. However, it is not known if this level of inhibition over the duration of a clinical trial will be sufficient. Moreover, it is not known if this level of inhibition will similarly be effective in iPD patients who do not have an activating mutation but have elevated LRRK2 kinase activity. Further compounding this is that the field still does not have a good way to assess CNS LRRK2 target engagement in the clinic. For example efforts to develop a LRRK2 PET tracer or a CSF biomarker have largely been unsuccessful with exception of the recently described CSF assay for assessing total LRRK2 levels^122^. This lack of understanding around the level of CNS target engagement presents a significant challenge when designing clinical trials for LRRK2 kinase inhibitors where, given the number of subjects required for adequate statistical power, challenges with patient requirement (e.g. LRRK2 G2019S mutation carriers) and potential cost, the number of dose levels of a given compound that can be practically tested in the clinic may be limited, potentially to only a single dose level vs placebo. In the case where an IC_50_ level of inhibition were to be tested, a negative outcome in the clinic could be interpreted as a need to push to higher levels of target engagement which would require additional clinical trials. One approach to mitigate this challenge could be target higher levels of LRRK2 kinase inhibition from the outset, i.e. IC_75_ or greater, however, this strategy increases the potential risk of running into treatment emergent adverse events which may impact the overall outcome of the study. Also, given that if LRRK2 therapeutics do modify the course of PD, and would therefore presumably be used for a lifetime after diagnosis, the long-term safety of the specific intervention will be of utmost importance. Based on studies in rodents and non-human primates, and the fact that humans with LRRK2 loss of function mutations are not known to have deleterious health effects^123^, blockade of LRRK2 kinase activity seems to be reasonably safe, although this remains an area of active investigation. Ultimately, the decision to move forward or not will depend on an informed collaboration between biopharma, regulatory agencies and people with PD.

## Conclusion

The LRRK2 field is at an exciting juncture with studies on the biology of this protein that can rely on two decades of groundwork that is increasingly verified and robustly built upon, resulting in clinical trials with LRRK2 targeting agents that are in their early phases. While the temptation may exist to leave the fundamental biology work for what it is and wait out the results of clinical trials that may confirm new clinical solutions for patients, history tells us that work on the fundamental biology of this target is more important than ever in order to be poised to interpret clinical data and prepare the next generation of targeting strategies. We know that a clinical trial may fail with only a small portion of responders, however this may provide the opportunity to work out why a small number respond and others do not.

## Data Availability

Data sharing is not applicable to this article as no datasets were generated or analysed during the current study.
